# International collaboration to improve physiotherapists’ training, Viet Nam

**DOI:** 10.2471/BLT.22.288100

**Published:** 2022-09-02

**Authors:** Didier Demey, Sidy OA Dieye, Veronique Feipel, Liz A Holey, Jonathon Kruger, Van Thanh Le, Prue Morgan, Margot A Skinner, Patrick Willems

**Affiliations:** a Handicap International, 138 Av. des Frères Lumière, Lyon, 69008, France.; b World Physiotherapy, London, England.; c Faculté des Sciences de la Motricité, Université Libre de Bruxelles, Brussels, Belgium.; d Leeds, England.; e Department of Physiotherapy, University of Medicine and Pharmacy, Ho Chi Minh City, Viet Nam.; f Department of Physiotherapy, Monash University, Melbourne, Australia.; g School of Physiotherapy, University of Otago, Dunedin, New Zealand.; h Faculté des Sciences de la Motricité, Université Catholique de Louvain, Louvain, Belgium.

## Abstract

**Problem:**

Like most low- and middle-income countries, Viet Nam has a scarcity of rehabilitation professionals and lacks training programmes that meet international standards.

**Approach:**

In 2018, four Vietnamese medical universities, the Université Catholique de Louvain, the Université Libre de Bruxelles, the Humanity & Inclusion charity and World Physiotherapy agreed to collaborate on strengthening pre-service education for physiotherapists in the country.

**Local setting:**

Viet Nam has a favourable environment for nurturing rehabilitation services and education: development funds have been available; government investment is increasing; and rehabilitation education has existed for many decades.

**Relevant changes:**

The collaboration resulted in the establishment of: (i) a 4-year, competency-based, entry-level curriculum for physiotherapists (bachelor’s degree); (ii) opportunities for continuing professional development; (iii) a 2-year master’s programme for physiotherapy lecturers and clinical supervisors; and (iv) a national physiotherapy association. In addition, four students were supported in studying for PhD degrees. Strong collaboration and comprehensive and complementary interventions have laid the foundations for sustainable, high-quality, educational programmes for physiotherapists, which will improve access to, and the standard of, rehabilitation services in Viet Nam, thereby leading to better patient outcomes.

**Lessons learnt:**

Curricula for entry-level physiotherapy programmes should be competency-based, be actively managed by national educators and meet international standards while being responsive to local priorities. To strengthen the rehabilitation workforce, educators involved in teaching and supervising training programmes should have the skills and knowledge required. A national professional physiotherapy association should be established to provide continuing professional development for physiotherapists and to take part in international collaborations.

## Résumé

**Problème:**

À l'instar de la plupart des pays à revenu faible et intermédiaire, le Vietnam est confronté à une pénurie de professionnels de la réadaptation et manque de programmes d’enseignement conformes aux normes internationales.

**Approche:**

En 2018, quatre facultés de médecine vietnamiennes ainsi que l'Université Catholique de Louvain, l'Université Libre de Bruxelles, l'association caritative Humanity & Inclusion et l'organisation World Physiotherapy ont décidé de collaborer afin de renforcer la formation initiale des kinésithérapeutes dans le pays.

**Environnement local:**

Le Vietnam présente des conditions propices au développement de formations et services de réadaptation: des fonds ont été débloqués; le gouvernement accroît ses investissements; et la réadaptation est enseignée depuis des décennies.

**Changements significatifs:**

La collaboration mise en place a permis de créer: (i) des études de kinésithérapie en quatre ans, fondées sur les compétences et accessibles aux débutants (diplôme de baccalauréat/licence); (ii) des opportunités d'évolution professionnelle continue; (iii) un programme de master en deux ans pour les professeurs de kinésithérapie et les superviseurs cliniques; et enfin, (iv) une association nationale de kinésithérapie. En outre, quatre étudiants ont bénéficié d'une aide pour poursuivre un doctorat. Cette étroite collaboration, accompagnée d'interventions globales et complémentaires, a jeté les bases d'un programme d'enseignement durable et de haut niveau pour les futurs kinésithérapeutes, améliorant par la même occasion la qualité et l'accès aux services de réadaptation au Vietnam et, par conséquent, les résultats observés chez les patients.

**Leçons tirées:**

Les études de kinésithérapie destinées aux débutants doivent reposer sur l'acquisition de compétences, être activement gérées par des professeurs nationaux et répondre aux normes internationales tout en tenant compte des priorités locales. Les enseignants impliqués chargés des cours et de la supervision des programmes de formation doivent posséder les connaissances et aptitudes requises pour consolider les équipes de réadaptation. Une association nationale de kinésithérapie doit voir le jour afin de proposer aux kinésithérapeutes une évolution professionnelle continue et d'établir des partenariats dans le monde entier.

## Resumen

**Situación:**

Al igual que la mayoría de los países de ingresos bajos y medios, Vietnam tiene un déficit de profesionales de la rehabilitación y carece de programas de formación que cumplan los estándares internacionales.

**Enfoque:**

En 2018, cuatro universidades de medicina vietnamitas, la Université Catholique de Louvain, la Université Libre de Bruxelles, la organización benéfica Humanity & Inclusion y World Physiotherapy acordaron colaborar en el fortalecimiento de la educación previa al servicio de los fisioterapeutas en el país.

**Marco regional:**

Vietnam cuenta con un entorno favorable para fomentar los servicios y la educación en materia de rehabilitación: se ha dispuesto de fondos para el desarrollo; la inversión del gobierno está aumentando; y la educación en materia de rehabilitación existe desde hace muchas décadas.

**Cambios importantes:**

La colaboración dio lugar a la creación de: (i) un plan de estudios inicial de 4 años, basado en competencias, para fisioterapeutas (licenciatura); (ii) oportunidades de desarrollo profesional continuo; (iii) un programa de máster de 2 años para profesores de fisioterapia y supervisores clínicos; y (iv) una asociación nacional de fisioterapia. Además, se apoyó a cuatro estudiantes para que cursaran un doctorado. La fuerte colaboración y las intervenciones integrales y complementarias han sentado las bases de unos programas educativos sostenibles y de alta calidad para fisioterapeutas, que mejorarán el acceso a los servicios de rehabilitación en Vietnam y el nivel de estos, lo que redundará en mejores resultados para los pacientes.

**Lecciones aprendidas:**

Los planes de estudio de los programas de fisioterapia de nivel inicial deben centrarse en las competencias, ser gestionados activamente por los educadores nacionales y cumplir los estándares internacionales, al tiempo que responden a las prioridades locales. Para reforzar el personal de rehabilitación, los educadores que participan en la enseñanza y la supervisión de los programas de formación deben tener las competencias y los conocimientos pertinentes. Se debería crear una asociación nacional de fisioterapia profesional para ofrecer un desarrollo profesional continuo a los fisioterapeutas y participar en colaboraciones internacionales.

## ملخص

**المشكلة:**

كما هو الحال في معظم الدول ذات الدخل المنخفض والمتوسط، فإن فييت نام تعاني من الندرة في أخصائيي إعادة التأهيل، وتفتقر إلى برامج التدريب التي تلبي المعايير الدولية.

**الأسلوب:**

في عام 2018، قامت أربع جامعات طبية فييت نامية، وهي Université Catholique de Louvain (الجامعة الكاثوليكية في لوفان)، وUniversité Libre de Bruxelles (جامعة بروكسيل الحرة)، ومؤسسة Humanity & Inclusion (الإنسانية والشمول) الخيرية، وWorld Physiotherapy (العلاج الفيزيائي العالمي)، بالاتفاق على التعاون في تعزيز تعليم ما قبل الخدمة لأخصائيي العلاج الطبيعي في البلاد.

**المواقع المحلية:**

تتمتع فييت نام ببيئة ملائمة للاهتمام بخدمات إعادة التأهيل والتعليم: حيث كانت تمويلات التنمية متوفرة؛ وكان الاستثمار الحكومي مستمرًا في التزايد؛ واستمرت إعادة التأهيل لعدة عقود.

**التغيّرات ذات الصلة:**

أدى التعاون إلى إنشاء: (1) منهج للمبتدئين لمدة 4 أعوام، قائم على الكفاءة لأخصائيي العلاج الطبيعي (درجة البكالوريوس)؛ و(2) فرص للتطوير المهني المستمر؛ و(3) برنامج للماجستير لمدة عامين لمحاضري العلاج الطبيعي والمشرفين السريريين؛ و(4) جمعية وطنية للعلاج الطبيعي. بالإضافة إلى ذلك، تم دعم أربعة طلاب في الدراسة للحصول على درجة الدكتوراه. أدى التعاون القوي والتدخلات الشاملة والتكميلية لوضع الأسس لبرامج تعليمية مستدامة وعالية الجودة لأخصائيي العلاج الطبيعي، وهو مما سيحسن الحصول على خدمات إعادة التأهيل، ويطور من مستواها في فييت نام، مما يؤدي إلى نتائج أفضل للمرضى.

**الدروس المستفادة:**

يجب أن تكون مناهج برامج العلاج الطبيعي للمبتدئين قائمة على الكفاءة، وأن تتم إدارتها بنشاط بواسطة المعلمين الوطنيين، وتفي بالمعايير الدولية مع الاستجابة للأولويات المحلية. لتعزيز القوى العاملة في مجال إعادة التأهيل، فإن المعلمين المشاركين في تدريس برامج التدريب والإشراف عليها، يجب أن يتمتعوا بالمهارات والمعرفة المطلوبة. يجب إنشاء جمعية وطنية مهنية للعلاج الطبيعي لتقديم التطوير المهني المستمر لأخصائيي العلاج الطبيعي، وللمشاركة في عمليات التعاون الدولي.

## 摘要

**问题:**

与大多中低收入国家一样，越南缺少康复治疗师以及符合国际标准的培训计划。

**方法:**

2018 年，四所越南医科大学、鲁汶大学、布鲁塞尔自由大学、人文与包容慈善机构和世界物理治疗联盟展开合作，以加强该国物理治疗师的职前教育。

**当地状况:**

越南拥有促进康复服务和教育的有利环境：发展基金已到位；政府正在增加投资；并且康复教育已开展数十年。

**相关变化:**

合作成果包括：(i) 为物理治疗师开设以能力为本位的四年制入门级课程（学士学位）；(ii) 提供继续职业发展的机会；(iii) 为物理治疗讲师和临床监护者开设两年制硕士课程；以及 (iv) 成立国家物理治疗协会。此外，该项目还资助四名学生攻读博士学位。强有力的合作以及全面和互补的干预措施为面向物理治疗师开展可持续和高质量的教育计划奠定了基础，这将改善越南康复治疗服务的可及性和标准，从而使患者得到更好的治疗效果。

**经验教训:**

物理治疗计划的入门课程应以能力为本位，由国家教育工作者积极管理，并在积极响应当地优先事项的同时符合国际标准。为了增强康复治疗人员的专业素养，参与教学和管理培训计划的教育工作者应具备所需的技能和知识。应成立国家专业物理治疗协会，为物理治疗师提供继续职业发展以及参加国际合作的机会。

## Резюме

**Проблема:**

Как и в большинстве стран с низким и средним уровнем дохода, во Вьетнаме не хватает реабилитологов и программ обучения, отвечающих международным стандартам.

**Подход:**

В 2018 году четыре медицинских университета Вьетнама (Лувенский католический университет, Брюссельский свободный университет, благотворительная организация Humanity & Inclusion, а также организация World Physiotherapy) договорились о сотрудничестве в области повышения дослужебного образования физиотерапевтов в стране.

**Местные условия:**

Вьетнам имеет благоприятную среду для развития услуг и образования в области реабилитации: наличие средств на развитие, увеличение государственных инвестиций и существование реабилитационного образования уже на протяжении многих десятилетий.

**Осуществленные перемены:**

Результатом сотрудничества стало создание: (i) 4-летней учебной программы начального уровня, ориентированной на формирование профессиональных навыков для физиотерапевтов (степень бакалавра); (ii) возможностей для постоянного профессионального развития; (iii) 2-летней магистерской программы для преподавателей физиотерапии и руководителей клиник; (iv) национальной ассоциации физиотерапевтов. Кроме того, четырем студентам помогли обучаться в докторантуре. Тесное сотрудничество и всесторонние, взаимодополняющие меры заложили основу для устойчивых, высококачественных образовательных программ для физиотерапевтов, которые улучшат доступ к реабилитационным услугам во Вьетнаме и повысят их уровень, что приведет к улучшению результатов лечения пациентов.

**Выводы:**

Учебные программы физиотерапии начального уровня должны основываться на формировании профессиональных навыков, активно управляться национальными преподавателями и соответствовать международным стандартам с учетом местных приоритетов. Чтобы усилить кадры в сфере реабилитационных услуг, преподаватели, участвующие в обучении и контроле учебных программ, должны обладать необходимыми навыками и знаниями. Необходимо создать национальную профессиональную физиотерапевтическую ассоциацию для обеспечения непрерывного профессионального развития физиотерапевтов и участия в международном сотрудничестве.

## Introduction

Around 7% of the Vietnamese population has a disability,^1^ which corresponds to more than 6 million individuals. In addition, the number of people with noncommunicable diseases in the country is rising,^2^ the population is ageing rapidly and Viet Nam has a road traffic crash rate above the average for South-East Asia.^3^^,^^4^ As a result, the need for rehabilitation services is increasing.^5^

In 2019, a systematic assessment of rehabilitation in Viet Nam found that most health facilities were equipped to provide rehabilitation services: the proportion ranged from 75% at the district level to 100% at the central level (unpublished data, Viet Nam Ministry of Health, 2020). However, only 7200 individuals had a pre-service training qualification in rehabilitation; the majority were physiotherapists with 2-year diploma certificates. Other qualified professionals included prosthetists, orthotists, speech language therapists and occupational therapists. In addition, a range of other health workers had undertaken short orientation courses in rehabilitation of 3 to 12 months’ duration. Overall, the quality of health and rehabilitation services provided across Viet Nam and patient outcomes were variable because of the low number of health-care professionals for the population size and the short duration of training.

Shortages of rehabilitation professionals, health-care workers with a relatively low level of skills and knowledge, and the inequitable distribution of these workers have been identified as major barriers to accessing rehabilitation services worldwide, particularly in low- and middle-income countries.^6^

Both pre-service training and continuing professional development are crucial for ensuring high-quality services.^7^ A 2017 report from the World Health Organization, entitled *Rehabilitation 2030: a call for action*,^8^ identified building an adequate, well-trained and multi-occupation rehabilitation workforce as central to strengthening rehabilitation in the health system. When faced with rehabilitation workforce shortages, organizations and individuals aiming to strengthen services and develop pre-service or initial training are usually presented with two options: (i) develop or strengthen high-quality, university-level, educational programmes aligned with international standards and requirements and having a long-term vision;^9^ or (ii) address urgent needs through short courses, such as orientation courses for other health-care providers (e.g. nurses, traditional doctors or community health workers), with the objective of enabling task-shifting. Although both approaches can lead to successful outcomes,^10^ the choice usually depends on the context and opportunities locally; funding, the time available, and local resources and expertise. These factors often make it less attractive to choose the first option even if it would be expected to have a more sustainable long-term impact.^6^^,^^11^

Here we describe how an international collaboration focused on the first option to strengthen pre-service education for physiotherapy with the goal of improving rehabilitation workforce and access to rehabilitation services in Viet Nam.

## Local setting

Over recent decades, rehabilitation services in Viet Nam have developed rapidly thanks to a favourable environment. First, international development funds have been available, which has enabled a variety of actors to support rehabilitation services. For example, since 1987 the United States Agency for International Development has contributed over 100 million United States dollars (US$) to support people with disabilities.^12^ Second, rehabilitation education (mostly for physiotherapists, prosthetists and orthotists) has existed in the country for almost 50 years. Third, a rehabilitation professional association (the Viet Nam Rehabilitation Association) was established in 1991 and had around 4000 members in 2022. Fourth, steady economic growth has enabled government investment in health and rehabilitation to increase: health-care expenditure in Viet Nam accounted for 7.5% of the country’s gross domestic product in 2017 and social health insurance covers an array of rehabilitation services.^13^^,^^14^ Finally, local authorities have developed several rehabilitation policy documents (e.g. the National Plan for Rehabilitation Services Development),^15^ which provide a solid foundation for building rehabilitation services. Still, much remains to be done, particularly with regard to workforce development and education.

## Approach

In Viet Nam, the disability charity Humanity & Inclusion, the Université Catholique de Louvain, the Université Libre de Bruxelles and World Physiotherapy collaborated with four Vietnamese medical universities (Hai Duong Medical Technology University, Da Nang University of Medical Technology and Pharmacy, the University of Medicine and Pharmacy at Ho Chi Minh City and Hong Bang International University) and the newly formed Viet Nam Physical Therapy Association to increase the capacities of the rehabilitation workforce by strengthening university-level and competency-based pre-service educational programmes for physiotherapists. At the same time, other organizations were involved in providing short courses. 

In 2016, Humanity & Inclusion and World Physiotherapy undertook a gap analysis of entry-level physiotherapy educational programmes at the four Vietnamese medical universities (Humanity & Inclusion and World Physiotherapy, unpublished data, 2017). Their main findings included the following: (i) existing physiotherapy curricula were not aligned with international standards and were not evidence-based; (ii) clinical placements did not promote independent clinical reasoning; and (iii) students were supervised by clinicians who lacked skills in contemporary patient management. On the basis of these findings, World Physiotherapy recommended: (i) developing a new national physiotherapy curriculum based on a World Physiotherapy education framework that is customized to meet the social, cultural and national priorities of Viet Nam;^16^ (ii) helping lecturers and clinical supervisors attain higher degrees that will enable them to deliver the curriculum and provide high-quality leadership; (iii) developing continuing professional education for physiotherapists, particularly those working in academia and clinical practice, to enable them to deliver the curriculum and provide clinical care to contemporary standards; (iv) developing national physiotherapy standards; and (v) supporting the establishment of a national physiotherapy association.

At the time, the Université Catholique de Louvain was already actively supporting physiotherapists in Viet Nam by providing staff to teach regular, short, training courses for physiotherapists and lecturers at the University of Medicine and Pharmacy at Ho Chi Minh City.

**Fig. 1:**
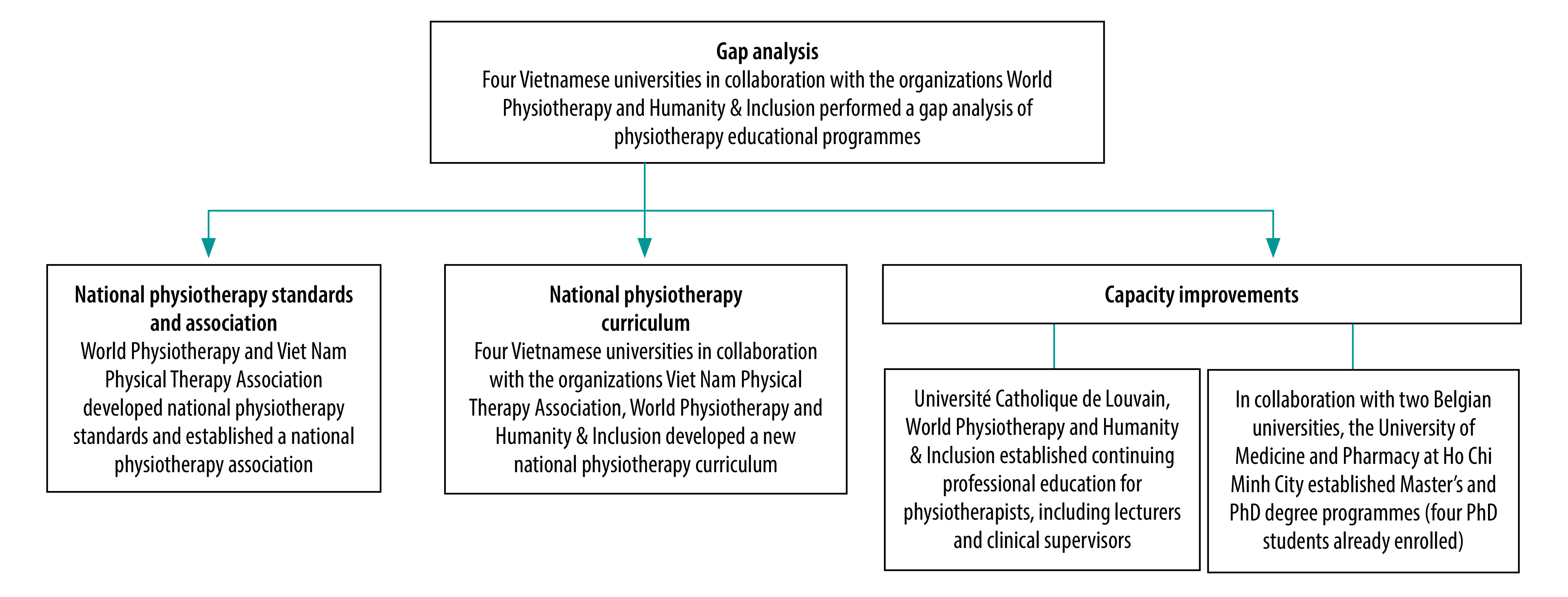
Activities of the collaboration for improving rehabilitation services, Viet Nam, 2016–2022

Notes: The four Vietnamese universities are Da Nang University of Medical Technology and Pharmacy, Hong Bang International University, University of Medicine and Pharmacy at Ho Chi Minh City and Hai Duong Medical Technology University. The two Belgian universities are Université Libre de Bruxelles and Université Catholique de Louvain.

The Viet Nam Physical Therapy Association was formally recognized as a member of World Physiotherapy in 2021. Meanwhile, the four Vietnamese medical universities, the Viet Nam Physical Therapy Association, World Physiotherapy and Humanity & Inclusion (with the support of the Vietnamese health ministry and education and training ministry and with funding from the United States Agency for International Development and the Australian Physiotherapy Association) started to develop national physiotherapist competency standards and a national curriculum framework for physiotherapists (Fig. 1). Then, World Physiotherapy mentors worked with each of the four universities to develop their own curricula, which were based on agreed competency thresholds and international standards. The curriculum development process was collaborative and fully involved university staff.

To address weaknesses in the educational level of staff involved in teaching physiotherapy, the Université Catholique de Louvain and the Université Libre de Bruxelles secured funds from the ARES federation^17^ to develop and implement a 2-year master’s degree programme that prioritized physiotherapy lecturers and student clinical supervisors. The programme was jointly developed and implemented by representatives of the Université Catholique de Louvain and the University of Medicine and Pharmacy at Ho Chi Minh City (with the support of World Physiotherapy and Humanity & Inclusion) and was approved by local academic authorities. In addition, funding was secured to enable four physiotherapy teachers from the University of Medicine and Pharmacy at Ho Chi Minh City to complete PhD degrees in Belgium. The Université Catholique de Louvain, Humanity & Inclusion and World Physiotherapy have maintained their involvement in developing continuing professional development courses for lecturers and clinical supervisors as well as for the wider physiotherapy profession.

Due to the coronavirus disease 2019 pandemic, from March 2020, the country closed its borders for more than 18 months. Teachers and consultants were prevented from entering Viet Nam as planned. To maintain momentum, hybrid curriculum development workshops were organized with Vietnamese teachers gathering at one university and consultants available online. Mentoring for individual universities was provided remotely. The master’s programme was adapted so that teaching could be temporarily provided online. When Viet Nam reopened its borders, travel and onsite workshops recommenced.

## Relevant changes

By early 2022, our combined interventions in Viet Nam had enabled collaborators to achieve the following core results. First, national competency standards for physiotherapists were developed and will soon be validated by the health ministry. Second, the Viet Nam Physical Therapy Association has 753 members and is one of the fastest growing World Physiotherapy member organizations. Third, the 4-year bachelor’s degree curricula have been revised for all four partner universities and have been reshaped to align with new competencies. These were due to be introduced in the 2022 to 2023 academic year. Fourth, the first cohort on the physiotherapist master’s programme started in late 2020 – 16 participants were expected to graduate in November 2022. A second cohort was planned to start shortly afterwards. Finally, three of the four PhD candidates from the University of Medicine and Pharmacy at Ho Chi Minh City had started work on their theses. The fourth candidate was due to start his thesis in late 2022.

## Lessons learnt

The three main lessons learnt during the project are summarized in Box 1. By ensuring these skills are core competencies to be acquired in bachelor’s or master’s degree programmes, our intervention is expected to have a long-lasting, positive impact on the quality of rehabilitation services and on patient outcomes.

Box 1 **Summary of main lessons learnt**

- Curricula for entry-level physiotherapy programmes should be competency-based and developed with the participation of national and international academics, and the national rehabilitation professional association should both meet international standards and be responsive to local priorities.
- To be effective, efforts to strengthen the rehabilitation workforce should focus on the curriculum for initial physiotherapy training. In addition, the capacities of teachers, lecturers and clinical supervisors should be improved to enable them to implement an up-to-date curriculum.
- Building a national professional physiotherapy association is important for achieving national and international recognition of the profession and for ensuring access to continuing professional development programmes.

Although significant investments were made at the start of our collaborative project (e.g. the United States Agency for International Development invested about US$ 150 000 in the education gap analysis and bachelor’s programme review, the ARES federation invested about US$ 500 000 to support the master’s and PhD programmes, and the Australian Physiotherapy Association invested US$ 30 000 to support the development of national competencies standards), our approach guarantees long-term sustainability. First, bachelor’s degree programmes have been revised with the participation of Vietnamese organizations, thereby ensuring local ownership. Second, universities and national authorities in Viet Nam are in the process of approving these new programmes. Third, the competence of existing teachers and clinical supervisors is being strengthened by participation in master’s or PhD programmes, which will ultimately enable them to assume responsibility for local programme administration.

We acknowledge that Viet Nam benefits from an environment that favours the nurturing of rehabilitation services and education and that many low-income and, even, middle-income countries may not have a similar advantage. Nevertheless, the collaborative approach we developed and implemented in Viet Nam and lessons learnt could be applied successfully in other countries.

## References

[1] Vietnam national survey on people with disabilities. Hanoi: General Statistics Office; 2018. Available from: https://www.gso.gov.vn/en/data-and-statistics/2019/03/vietnam-national-survey-on-people-with-disabilities-2016/ [cited 2022 Feb 12].

[2] Joint mission of the United Nations Interagency Task Force on the Prevention and Control of Noncommunicable Diseases. Vietnam, 12–16 September 2016. WHO/NMH/NMA/17.88. Geneva: World Health Organization; 2017. Available from: https://apps.who.int/iris/bitstream/handle/10665/332125/WHO-NMH-NMA-17.88-eng.pdf [cited 2022 Aug 12].

[3] Workshop to launch the report “Towards a comprehensive national policy for an ageing in Viet Nam”. Hanoi: United Nations Population Fund Viet Nam; 2019. Available from: https://vietnam.unfpa.org/en/news/workshop-launch-report-%E2%80%9Ctowards-comprehensive-national-policy-ageing-viet-nam%E2%80%9D [cited 2022 Aug 12].

[4] Marukatat S. Thailand tops ASEAN road death table. Bangkok Post. 2018 Dec 7. Available from https://www.bangkokpost.com/thailand/general/1589682/thailand-tops-asean-road-death-table [cited 2022 Aug 12].

[5] Jesus TS, Landry MD, Brooks D, Hoenig H. Physical rehabilitation needs per condition type: results from the Global Burden of Disease study 2017. Arch Phys Med Rehabil. 2020 Jun;101(6):960–8. 10.1016/j.apmr.2019.12.020 32035140

[6] Rehabilitation competency framework. Geneva: World Health Organization; 2020. Available from: https://www.who.int/publications/i/item/9789240008281 [cited 2022 Jan 25].

[7] Khan F, Amatya B, de Groote W, Owolabi M, Syed IM, Hajjoui A, et al. Capacity-building in clinical skills of rehabilitation workforce in low- and middle-income countries. J Rehabil Med. 2018 May 8;50(5):472–9. 10.2340/16501977-2313 29487941

[8] Rehabilitation 2030: a call for action. Geneva: World Health Organization; 2017. Available from: https://www.who.int/publications/m/item/rehabilitation-2030-a-call-for-action [cited 2022 Aug 12].

[9] Education. Policy statement. London: World Physiotherapy; 2019. Available from: https://world.physio/sites/default/files/2020-04/PS-2019-Education.pdf [cited 2021 Dec 17].

[10] Nesbit KC, Clark A. Rehabilitation training for community health workers: a five-year study. Int J Health Promot Educ. 2019;57(1):3–12. 10.1080/14635240.2018.1538808

[11] Strengthening human resources for rehabilitation in Tajikistan. Copenhagen: World Health Organization Regional Office for Europe; 2018. Available from: https://www.who.int/news-room/feature-stories/detail/strengthening-human-resources-for-rehabilitation-in-tajikistan [cited 2022 Feb 23].

[12] Vietnam. Persons with disabilities. Washington, DC: United States Agency for International Development; 2022. Available from: https://www.usaid.gov/vietnam/persons-with-disabilities [cited 2022 Feb 12].

[13] Das K. Vietnam: growing demand for healthcare services. Vietnam Briefing. 2018 Sep 14. Available from: https://www.vietnam-briefing.com/news/vietnam-growing-demand-healthcare-services.html/ [cited 2022 Feb 12].

[14] [On regulation for rehabilitation techniques and items and reimbursement of daytime rehabilitation costs under coverage of health insurance funds]. Circular 18/2016/TT-BYT. Hanoi: Viet Nam Ministry of Health; 2016. Vietnamese. Available from: https://thuvienphapluat.vn/van-ban/Bao-hiem/Thong-tu-18-2016-TT-BYT-danh-muc-ky-thuat-vat-tu-y-te-phuc-hoi-chuc-nang-ban-ngay-pham-vi-thanh-toan-318971.aspx [cited 2022 Feb 12].

[15] Approving the national plan for rehabilitation service development for the 2014–2020 period. 4039/QD-BYT. Hanoi: Vietnam Ministry of Health; 2014. Available from: https://vanbanphapluat.co/decision-4039-qd-byt-2014-approving-the-national-plan-for-rehabilitation-service-development [cited 2022 Aug 12].

[16] Physiotherapist education framework. London: World Physiotherapy; 2011. Available from: https://world.physio/sites/default/files/2021-07/Physiotherapist-education-framework-FINAL.pdf [cited 2021 Dec 17].

[17] L’Académie de recherche et d’enseignement supérieur (ARES) [internet]. Brussels: L’Académie de recherche et d’enseignement supérieur; 2022. French. Available from: https://www.ares-ac.be/fr/ [cited 2022 Aug 12].

